# Tumor-Induced Osteomalacia Caused by Primary Fibroblast Growth Factor 23 Secreting Neoplasm in Axial Skeleton: A Case Report

**DOI:** 10.1155/2012/185454

**Published:** 2012-12-17

**Authors:** Gunjan Y. Gandhi, Aashish A. Shah, Kevin J. Wu, Vivek Gupta, Ali Reza Shoraka

**Affiliations:** ^1^Division of Endocrinology, Department of Internal Medicine, Mayo Clinic, 4500 San Pablo Road, Jacksonville, FL 32224, USA; ^2^Department of Pathology, Mayo Clinic, 4500 San Pablo Road, Jacksonville, FL 32224, USA; ^3^Department of Radiology, Mayo Clinic, 4500 San Pablo Road, Jacksonville, FL 32224, USA

## Abstract

We report the case of a 66-year-old woman with tumor-induced osteomalacia (TIO) caused by fibroblast growth factor 23 (FGF-23) secreting mesenchymal tumor localized in a lumbar vertebra and review other cases localized to the axial skeleton. She presented with nontraumatic low back pain and spontaneous bilateral femur fractures. Laboratory testing was remarkable for low serum phosphorus, phosphaturia, and significantly elevated serum FGF-23 level. Magnetic resonance imaging (MRI) of the lumbar spine showed a focal lesion in the L-4 vertebra which was hypermetabolic on positron emission tomography (PET) scan. A computed tomography (CT) guided needle biopsy showed a low grade spindle cell neoplasm with positive FGF-23 mRNA expression by reverse transcriptase polymerase chain reaction (RT-PCR), confirming the diagnosis of a phosphaturic mesenchymal tumor mixed connective tissue variant (PMTMCT). The patient elected to have surgery involving anterior resection of L-4 vertebra with subsequent normalization of serum phosphorus. Including the present case, we identified 12 cases of neoplasms localized to spine causing TIO. To our knowledge, this paper represents the first documented case of lumbar vertebra PMT causing TIO. TIO is a rare metabolic bone disorder that carries a favorable prognosis. When a lesion is identifiable, surgical intervention is typically curative.

## 1. Introduction

Osteomalacia is a metabolic bone disorder characterized by impaired mineralization of osteoid matrix in mature bone. Acquired forms of osteomalacia include renal tubulopathy (Fanconi's type) and FGF-23 secreting PMTs; the latter referred to as TIO or oncogenic osteomalacia (OO). TIO is a rare paraneoplastic form of renal phosphate wasting that results in phosphaturia, a defect in vitamin D metabolism, and osteomalacia [1–3]. We report the case of a 66-year-old woman with TIO caused by a FGF-23 secreting mesenchymal tumor localized in the axial skeleton, more specifically in the fourth lumbar vertebra. Confirmation of PMTMCT was obtained by CT guided needle biopsy which revealed a low grade spindle cell neoplasm with positive FGF-23 mRNA expression by RT-PCR. The patient underwent surgery with removal of the tumor and subsequent normalization of biochemical abnormalities. Including the present case, we have identified only 12 cases of neoplasms localized to spine causing TIO. To our knowledge, this paper represents the first documented case of lumbar vertebra PMT causing TIO.

## 2. Case Report

**Figure 1:**
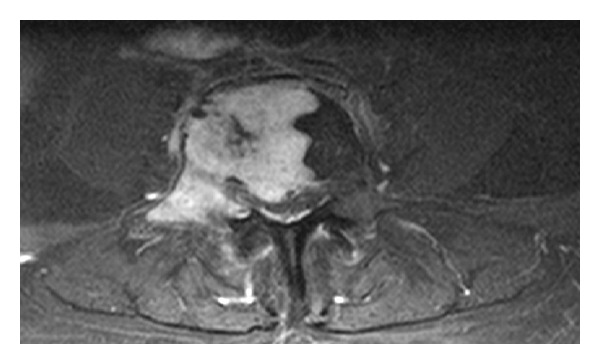
MRI lumbar spine: enhanced T1 weighted axial image. Enhancing, well-defined benign phosphaturic mesenchymal tumor in lumbar vertebra.

**Figure 2:**
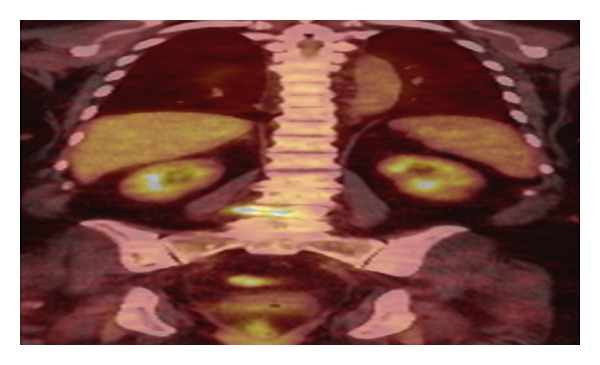
Coronal PET-CT scan. Hypermetabolic focus corresponding to the lumbar lesion.

**Figure 3:**
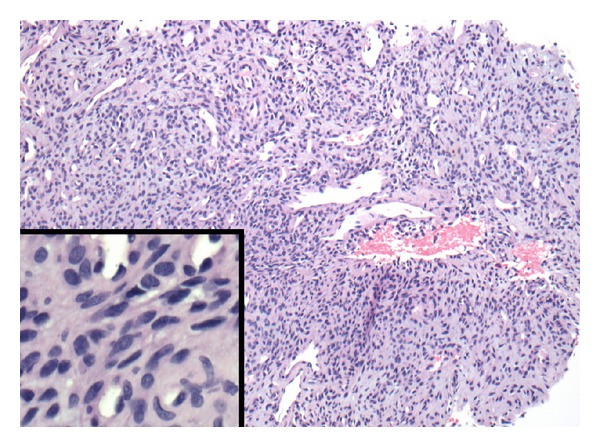
Photomicrograph (H&E stain, 10x; inset lower left, 60x) showing uniform bland appearing spindle cells with hemangiopericytoma-like vasculature. Rare multinucleate giant cells (not pictured) were also present.

A 66-year-old female presented with nontraumatic, chronic dull-aching low back pain with worsening symptoms for the previous one year. The pain affected her walking such that she was “waddling” to avoid the pain. On occasions the pain would radiate to the right leg as well as anterior and lateral thigh but not past the knee. There were no associated paresthesias or significant weakness. She denied history of cancer, weight loss, fever, chills, or night sweats. There was no history of nephrolithiasis or bone fractures. She was being treated for diabetes and hypertension. She had history of pulmonary emboli of unclear etiology in the 1990 s. Surgical history included cholecystectomy in 1994, uterine endometrial ablation in 1996, and bilateral carpal tunnel surgeries. She had an allergy to penicillin causing a rash and an intolerance to sulfa causing abdominal discomfort. The patient reported remote history of smoking for 15 years, quitting 25 years ago. Family history was negative for bone disease. Physical exam was remarkable for her being overweight. She had a side-to-side waddle in her gait and slightly lifted her feet on walking. Straight leg raising test was negative. She did not have tenderness to percussion of the lower back but did have a gibbous at L-4. There was no back pain with movement. She had weakness at the iliopsoas and quadriceps bilaterally but no loss of sensation. An outside MRI of the lumbar spine showed an infiltrative lesion in the L-4 vertebra with collapse of the body (confirmed on repeat MRI at our institution, Figure 1). CT scan confirmed a pathologic fracture of L-4 with a lytic lesion. A bone scan showed mildly increased radiotracer uptake at L-4, and additional uptake was seen in several lower thoracic ribs bilaterally, as well as the xiphoid process, upper right femur, and skull, suggesting metabolic bone disease. The L-4 lesion was hypermetabolic on PET scan (Figure 2). Initial CT guided biopsy of the lesion was nondiagnostic demonstrating only bone, fibrous tissue, and cartilage. Laboratory testing was remarkable for low serum phosphorus of 1.5 mg/dL (2.5–4.5 mg/dL). Additional abnormalities included elevated serum calcium of 10.6 mg/dL (8.9–10.1 mg/dL), inappropriately high parathyroid hormone (PTH) of 120 pg/mL (15–65 pg/mL), low 1,25-dihydroxyvitamin D of <8 pg/mL (18–78 pg/mL), and mildly elevated serum alkaline phosphatase of 162 u/L (45–115 u/L). Serum albumin was normal at 4.0 g/dL (3.5–5.0 g/dL), serum creatinine was 1.2 mg/dL (0.6–1.1 mg/dL), and 25-hydroxyvitamin D was normal at 31 ng/mL (25–80 ng/mL). She had frank phosphaturia, especially for her level of serum phosphorus (772 mg, normal 0–1,099 mg). 24-hour urine calcium was low at 10 mg (20–275 mg). Serum and urinary protein electrophoresis with immunofixation were normal. Thyroid stimulating hormone (TSH) was normal at 2.12 mIU/L (0.3–5.0 mIU/L). Parathyroid scan was normal. Plasma FGF-23 from a peripheral vein was significantly elevated at 3,500 RU/mL (<180 RU/mL), confirming TIO. Oral phosphate supplementation and calcitriol were prescribed. She was subsequently lost to followup from our medical center for two years, and when she returned, she reported that she had internal fixation with a rod placement for a spontaneous right femur fracture. She also had deep pain in her left thigh and was told by a local provider that she had a “brown tumor” in the left femur based on plain radiographs. She had not been taking phosphate supplements or calcitriol on a regular basis. Repeat laboratory investigations continued to show a significantly low phosphorus of 1.4 mg/dL (2.5–4.5 mg/dL), high serum calcium of 10.6 mg/dL (8.9–10.1 mg/dL), elevated PTH of 229.1 pg/mL (10–65 pg/mL), and serum creatinine of 0.9 mg/dL (0.6–1.1 mg/dL). Plasma FGF-23 level remained significantly elevated at 4,460 RU/mL (<180 RU/mL). A repeat CT guided needle biopsy of L-4 showed a low grade spindle cell neoplasm with positive FGF-23 mRNA expression by RT-PCR (Figure 3), confirming the diagnosis of mixed phosphaturic mesenchymal tumor (PMT). She suffered a spontaneous left peritrochanteric fracture requiring placement of an intramedullary device. The patient then elected to have surgery involving anterior resection of the L-4 vertebral body to complete total spondylectomy at this spinal level, along with corpectomy. Pathology of the resection specimen confirmed a spindle cell neoplasm consistent with PMTMCT. Six months after surgical resection, she was independent in activities of daily living but was using a walker for ambulation due to some difficulty walking and right foot drop. She was prescribed physical therapy. Follow-up laboratory testing revealed normal serum phosphorus at 2.5 mg/dL (2.5–4.5 mg/dL) and calcium of 9.6 mg/dL (8.9–10.1 mg/dL). PTH was 619 pg/mL (10–65 pg/mL). This is on minimal phosphate supplementation without calcitriol. Repeat plasma FGF-23 level remained elevated but had greatly decreased to 422 RU/mL (<180 RU/mL).

## 3. Discussion

TIO is a rare metabolic bone disorder that in most cases carries a favorable prognosis [1–3]. One of the earliest observations of a patient with tumor and osteomalacia was reported by McCance in 1947 [15] and further described by Prader et al. [16]. Adults often present with progressive myalgias, bone pain, and fatigue which are often followed by recurrent fractures. Children demonstrate inability to ambulate, growth stunting, and skeletal deformities. The average time from onset to a presumptive diagnosis is often more than 2.5 years due to underrecognition of the condition [3]. Definitive treatment is often further delayed by an average of another 5 years from diagnosis due to difficulty with tumor localization [3]. The metabolic effects are induced by tumoral elevation of FGF-23, which is related to the FGF-19 subfamily and normally secreted by osteocytes [17]. FGF-23 causes inhibition of both sodium-phosphate cotransport and vitamin D 1-alpha-hydroxylase in the renal tubule. Subsequently, hypophosphatemia, renal phosphate wasting (low tubular maximum for reabsorption of phosphorus per liter of glomerular filtrate (TmP/GFR)), and decreased 1,25-dihydroxyvitamin D levels ensue. PMTMCT is a rare bone and soft tissue neoplasm that is frequently associated with TIO due to secretion of FGF-23. While these tumors may demonstrate a spectrum of histologic features which may not be wholly demonstrated, especially in small biopsies, the typical microscopic appearance of PMTMCT is that of a spindle cell proliferation often associated with a myxochondroid matrix and calcifications. A vascular pattern that is reminiscent of hemangiopericytoma with staghorn like vessels may be present with variable numbers of multinucleate giant cells. The nuclear features tend to be low grade appearing. While PMTMCT frequently pursues a benign course, a minority of cases with sarcomatous histologic features and demonstrating clinically malignant behavior have been described [8]. Positive immunoreactivity for FGF-23, vimentin, and in some cases actin has been reported. Tumors causing TIO may be difficult to localize. Imaging modalities that are helpful to find tumors in common sites (extremities and craniofacial locations) include CT, MRI, PET-CT, octreotide, and sestamibi, as well as bone scintigraphy. Selective venous sampling with FGF-23 may also be helpful, especially when there is a suspicious lesion on imaging [18]. When a lesion is identifiable, surgical intervention is curative in the majority of cases. Complete tumor resection results in correction of biochemical abnormalities and remineralization of bone [19]. In the setting of unidentifiable tumor location and/or multiple comorbidities, patients are often nonsurgical candidates and may require long-term treatment with phosphorus (to replace ongoing renal phosphorus loss) and calcitriol (to supplement insufficient kidney production of 1,25-dihydroxyvitamin D). Potential complications of medical therapy include hypercalcemia, nephrocalcinosis, and kidney stone formation. In rare instances, octreotide has been used for treatment with some success as some mesenchymal tumors express somatostatin receptors that bind octreotide [20]. 

To our knowledge, this paper represents the first documented case of lumbar vertebra PMTMCT causing TIO. The tumors responsible for TIO can be found anywhere in the body; however, only 11 prior cases have been described in the spine (Table 1) [4–14]. Only three spinal TIO cases, including ours, utilized RT-PCR to identify tissue expression of FGF-23 [10, 13]. In the majority of these cases, either PMT or PMT mixed connective tissue variant (MCT) was diagnosed histologically. Other diagnoses included neuroendocrine tumor, plasmacytoma, osteosarcoma, and osteoblastoma. All cases, including ours, involved surgical intervention as a part of therapy. Chemo- and/or radiotherapy were used in less than half of spinal TIO cases [4, 6–8, 11]. In the present case, resection of tumor with spondylectomy using anterior retroperitoneal approach and L-4 corpectomy was curative. This entity should be considered as part of the differential diagnosis in adults with spontaneous bone fractures as well as unexplained bone pain. 

## Figures and Tables

**Table 1:** Characteristics of spinal cases of TIO.

Authors, year	Age (yrs), sex	Spinal level	Chemo- and/or radiotherapy	Histological diagnosis	RT-PCR used for FGF-23 tissue expression
(1) Stone et al., 1992 [4]	33, F	T 3-4	Yes	Neuroendocrine tumor	No
(2) Yu et al., 1995 [5]	58, F	C-2	No	PMT	No
(3) Terek and Nielsen, 2001 [6]	14, M	Sacrum	Yes	Osteosarcoma	No
(4) Boriani and Campanacci, 1978 [7]	18, M	Sacrum	Yes	Osteoblastoma	No
(5) Folpe et al., 2004 [8]	32, F	C-1	Yes	Malignant PMTMCT	No
(6) Pirola et al., 2009 [9]	57, M	T-4	No	PMT	No
(7) Mavrogenis et al., 2010 [10]	42, F	Sacrum	No	PMTMCT	Yes
(8) Chua et al., 2008 [11]	34, F	T-3	Yes	Plasmacytoma	No
(9) Sciubba et al., 2009 [12]	56, F	T-8	No	PMT	No
(10) Akhter et al., 2011 [13]	52, M	C-5	No	PMTMCT	Yes
(11) Marshall et al., 2010 [14]	55, F	T-12	No	PMTMCT	No
(12) Present case, 2012	66, F	L-4	No	PMTMCT	Yes

PMTMCT: phosphaturic mesenchymal tumor mixed connective tissue type.

PMT: phosphaturic mesenchymal tumor.

C: cervical, T: thoracic, L: lumbar.

F: female, M: male.

## Abbreviations

TIO: Tumor-inducedosteomalacia
FGF-23: Fibroblastgrowthfactor 23
MRI: Magneticresonanceimaging
PET: Positronemissiontomography
CT: Computedtomography
RT-PCR: Reversetranscriptase polymerasechainreaction
PMTMCT: Phosphaturicmesenchymaltumor mixedconnectivetissuevariant
OO: Oncogenicosteomalacia
TmP/GFR: Tubularmaximumforreabsorptionofphosphorusperliterofglomerularfiltrate
PTH: Parathyroidhormone
TSH: Thyroidstimulatinghormone
PMT: Phosphaturicmesenchymaltumor
MCT: Mixedconnectivetissue
C: Cervical
T: Thoracic
L: Lumbar
F: Female
M: Male.
